# Keratin 6, 16 and 17—Critical Barrier Alarmin Molecules in Skin Wounds and Psoriasis

**DOI:** 10.3390/cells8080807

**Published:** 2019-08-01

**Authors:** Xiaowei Zhang, Meimei Yin, Ling-juan Zhang

**Affiliations:** 1 School of Pharmaceutical Sciences, Xiamen University, Xiamen 361102, China; 2 Department of Dermatology, University of California, San Diego, La Jolla, CA 92093, USA

**Keywords:** keratins, epidermal keratinocytes, barrier alarmins, skin wounds, psoriasis, proliferation, innate immune responses, autoimmune

## Abstract

Located at the skin surface, keratinocytes (KCs) are constantly exposed to external stimuli and are the first responders to invading pathogens and injury. Upon skin injury, activated KCs secrete an array of alarmin molecules, providing a rapid and specific innate immune response against danger signals. However, dysregulation of the innate immune response of KCs may lead to uncontrolled inflammation and psoriasis pathogenesis. Keratins (KRT) are the major structural intermediate filament proteins in KCs and are expressed in a highly specific pattern at different differentiation stages of KCs. While KRT14-KRT5 is restricted to basal proliferative KCs, and KRT10-KRT1 is restricted to suprabasal differentiated KCs in normal skin epidermis, the wound proximal KCs downregulate KRT10-K1 and upregulate KRT16/KRT17-KRT6 upon skin injury. Recent studies have recognized KRT6/16/17 as key early barrier alarmins and upregulation of these keratins alters proliferation, cell adhesion, migration and inflammatory features of KCs, contributing to hyperproliferation and innate immune activation of KCs in response to an epidermal barrier breach, followed by the autoimmune activation of T cells that drives psoriasis. Here, we have reviewed how keratins are dysregulated during skin injury, their roles in wound repairs and in initiating the innate immune system and the subsequent autoimmune amplification that arises in psoriasis.

## 1. Introduction:

As the first physical and immunological barrier of the human body, the skin is a highly specialized organ composed of three primary layers: the outermost epidermis, the dermis and dermal white adipose tissue [1,2,3,4]. The epidermis, derived from a single layer of progenitor cells in embryos to a multilayered stratified epithelium during development, is at the front line of defense and is constantly exposed to a variety of environmental insults such as mechanical trauma, pathogens and chemical irritations [5,6,7]. As a result, keratinocytes, the most abundant cell type of the skin epidermis, has evolved to provide rapid and situation-specific innate immune responses upon sensing danger signals. During skin injury or infection, damage-associated molecular patterns (DAMPs) released by host necrotic cells or pathogen-associated molecular patterns (PAMPs) are recognized by keratinocytes through pattern recognition receptors (PRRs), leading to the rapid induction of “alarmins”, such as antimicrobial peptides, the S100 family of proteins and proinflammatory cytokines/chemokines that initiate a host innate immune defense against insults. These keratinocyte-derived alarmins not only directly attack invading pathogens, but can also attract or activate immune cells (such as dendritic cells, macrophages, neutrophils and T cells) and promote the development of adaptive immunity against pathogens or danger signals for long term protection.

Keratins (KRT), the major components of the epithelial cytoskeleton, are responsible for maintaining the structural stability and integrity of keratinocytes. So far, more than 54 mammalian keratins have been identified, contributing to ~30–80% of total protein and forming the ~10 nm intermediate filaments (IFs) in keratinocytes [7,8,9]. These highly diverse keratins are subdivided into two classes based on their pH: The acidic type I keratins (KRT9-KRT40) and the neutral–basic type II keratins (KRT1-KRT8) [10,11,12]. Keratins form a heterodimer between one type I keratin and one type II keratin, while self-assembling into antiparallel, staggered tetramers, forming an intermediate filament through longitudinal and lateral interaction [9]. Genes encoding type I and II keratins are clustered into two distinct chromosomal regions including chromosome17q12–q21 for type I keratins (except K18) and chromosome12q11–q13 for all type II keratins and KRT18 [13,14,15]. Despite their distinct locations on the genome, specific pairs of type I and type II keratins exhibit a highly specific and consistent expression pattern within a specific epidermal cell layer [11,16]. For example, in the interfollicular epidermis, KRT14-KRT5 is the major type I-type II keratin pair expressed in proliferative basal keratinocytes, whereas differentiated keratinocytes in the suprabasal layers downregulate KRT14-KRT5 and express KRT10-KRT1 as the major keratin pair. During wounding, stressed keratinocytes rapidly induce de novo transcription of KRT16/17-KRT6, whose expression is normally restricted to epidermal cells of glabrous skin, the oral mucosa, and several appendages [7].

Epidermal keratinocytes are constantly exposed to physical stress, and therefore a primary role of the keratin intermediate filament is to act as a flexible scaffold and provides resilience, enabling epidermal cells to resist mechanical stress [17]. Similar to other IFs, keratins have a head-rod-tail structure, with a central α-helical rod domain that is essential for intermediate filament formation [18,19]. Keratin IFs extend from the desmosomes to the nuclear membrane, providing tissue resilience to resist environmental stresses [20]. In addition to providing structural support to epithelial cells, growing evidence has shown that keratins also regulate cell proliferation, migration, adhesion and inflammatory features of keratinocytes [7,16,21]. Mutation or abnormal expression of keratin proteins is associated with a variety of skin diseases, such as skin blistering diseases (Epidermolysis bullosa simplex and Epidermolytic hyperkeratosis), psoriasis and skin tumors (squamous cell carcinoma and basal cell carcinoma). In this review, we have focused on the role of keratin proteins during skin wounding and how dysregulation of these keratins may lead to keratinocyte hyperproliferation and the development of an autoimmune amplification loop that drives the pathogenesis of psoriasis.

## 2. Keratin Expression During Skin Development and in Skin Diseases

**Figure 1:**
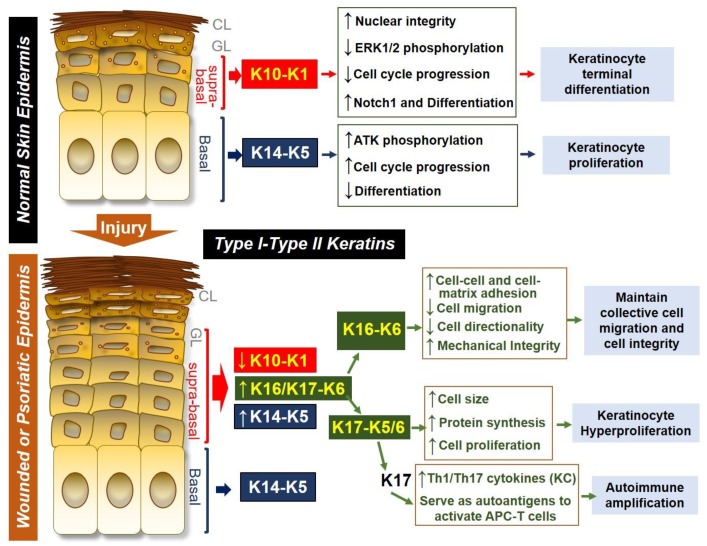
Keratin expression and function in normal skin epidermis or in wounded/psoriatic skin epidermis. In normal skin epidermis, basal keratinocytes express type I and type II keratin pair KRT14 (K14)-KRT5 (K5), which is necessary for maintaining keratinocyte proliferation by increasing AKT phosphorylation, promoting cell cycle progression and inhibiting spontaneous differentiation of keratinocytes. In contrast, cells located at the suprabasal epidermal layers express K10-K1 as the dominant keratin pair, and this keratin pair promotes terminal differentiation of keratinocytes by decreasing ERK1/2 phosphorylation, inhibiting cell cycle progression, and increasing Notch1 expression while maintaining nuclear integrity of differentiating cells. During skin injury or in psoriasis, suprabasal keratinocytes downregulate K10-K1, upregulate K14-K5 and strongly induce the expression of K16/K17-K6. The K16-K6 keratin pair functions to maintain collective cell migration and integrity by increasing cell-cell and cell-matrix contact, decreasing cell migration and directionality while maintaining mechanical integrity. K17 can pair with either K6 or K5 to drive keratinocyte hyperproliferation by increasing cell size and increasing protein synthesis. K17 can also induce Th1/Th17 cytokine production from keratinocytes, and K17 peptides can also serve as an autoantigen to promote antigen presenting cell (APC)-T cell activation, leading to autoimmune amplification during psoriasis pathogenesis.

Keratins have been widely used as marker proteins for various epithelial cell proliferation or differentiation stages as well as for epidermal disease diagnostics. In normal interfollicular skin, the expression of KRT14-KRT5 is considered to be the hallmark of basal keratinocytes including a cluster of progenitor cells, whereas the expression of KRT10-KRT1 reflects the early differentiation stage of keratinocytes (Figure 1). Furthermore, KRT16/17-KRT6 expression in keratinocytes represents a highly activated and proliferative stage under pathological conditions [7,22,23,24]. These keratin pairs collectively provide a flexible scaffold enabling keratinocytes to resist physical trauma and to regulate various functions including protection from apoptosis and the regulation of immune homeostasis.

### 2.1. Keratin Expression During Epidermal Development

The expression of keratins is tightly controlled at a time and cell–stage specific manner during epidermal development. At around embryonic day (E) 8.5, the progenitor cells express the keratin pair KRT18-KRT8, which is then gradually replaced by KRT14-KRT5 when the progenitor cells become specified to an epidermal cell fate starting around E9.5 [7,25,26]. From E10.5, keratinocytes from the single epidermal layer begin to move upward through a process of differentiation and stratification, forming the suprabasal layers, in which KRT14-KRT5 is replaced by KRT10-KRT1 [7]. From E16.5-E18/5, KCs at the suprabasal layers become terminally differentiated and continue to move upward forming the granular layers. These terminally differentiated cells downregulate KRT10-KRT1 transcript levels and in turn express late differentiation markers, such as Transglutaminase, Loricrin (LOR), Filaggrin (FLG) and Involucrin (INV). Finally, terminally differentiated cells become flattened, forming cornified envelops consisting of keratin proteins and acquire the barrier function before birth (~E19.5) [7,27]. In humans, epithelialization is not fully completed until ~34 weeks of gestation, leaving premature neonates susceptible to environmental insults and pathogens.

The expression patterns of keratin pairs are highly specific to the differentiation or activation stages of the epithelial cells, suggesting that each keratin pair is highly adapted for a specific function. The drastic KRT14-KRT5 to KRT10-KRT1 keratin pair switch during early keratinocyte differentiation was first recognized in the 1980s [28,29,30], and follow-up studies revealed distinct cellular functions of these two keratin pairs. Knockdown of KRT14 in keratinocytes led to decreased phosphorylated AKT levels and a substantial decrease in cell proliferation, along with an increase in the expression of keratinocyte differentiation markers [31]. In contrast, K10 deficiency in mice led to the activation of ERK1/2 kinase and epidermal hyperproliferation [32], and the forced expression of K10 in basal keratinocytes ceased cell cycle progression and promoted early differentiation of the cells [33]. While deletion of KRT10-KRT1 disrupted the desmosomal structure and nuclear integrity of epidermal cells, it did not impair epidermal stratification [34]. It was still not clear why these keratin pairs performed a diverse cellular function while sharing a common coiled-coil structure until the crystal structures of KRT14-KRT5 and KRT10-KRT1 were reported in recent years [35,36]. These studies have identified key structural differences that may shift the overall surface shape, charge, and hydrophobicity of K1-K10 compared to K5-K14, contributing to their distinct function in controlling proliferation and differentiation of keratinocytes.

### 2.2. Keratin Expression During Skin Wounding

Upon physical breach, skin resident keratinocytes, dermal fibroblasts along with various inflammatory immune cells cooperatively contribute to the orchestration of a train of cellular and molecular wound healing process, including four precisely programmed phases: hemostasis, inflammation, proliferation and remodeling [37,38]. In response to injury, keratinocytes are quickly activated and rapidly produce a variety of “alarmins” including antimicrobial peptides and proinflammatory cytokines that attract and/or activate immune cells to provide immediate as well as long term protection against a danger signal. During the wound healing process, the wound-proximal keratinocytes also transiently suspend terminal differentiation, and undergo dramatic changes in protein translation; in cell size and shape and in cell-cell contact and cell-matrix adhesiveness, preparing for active migration and proliferation during the healing phase.

KRT6, 16 and 17, which are normally not expressed in the interfollicular epidermis, have been recognized as barrier alarmins that are rapidly induced in keratinocytes upon wounding. The expression of the type II IFs KRT6a and KRT6b isoforms together with their type I partners KRT16 and KRT17 is rapidly and robustly induced in stressed keratinocytes at the suprabasal layers of the epidermis within hours after injury [39,40,41]. This induction of KRT6/16/17 occurs at the expense of the KRT10 and KRT1 keratin pair, which function to inhibit cell cycle progression and promote differentiation in normal KCs [42,43]. Expression of KRT6, 16 and 17 persist through the epithelial remodeling phases until the barrier function is restored, suggesting these keratins play important physiological roles during repair.

### 2.3. Keratin 6, 16 and 17 are Hallmarks of Psoriasis

Psoriasis is a chronic, recurrent autoimmune skin disease characterized by well-demarcated and raised areas of erythematous plaques, and is often covered by silvery scaling [44]. It is estimated that psoriasis is affecting ~3% of the U.S. and European populations, and ~0.5% of the Chinese population as well as other Asian countries [45]. Epidermal hyperproliferation, increased dermal vascularity and dermal leukocyte infiltration are the three histological features of psoriasis. However, the etiology of psoriasis remains obscure. Psoriasis is considered to be a T cell-mediated disease, whereby a mix of Th1 and Th17 T cell-derived cytokines, such as IFNγ, IL17A and IL22, drive the pathological hyperproliferation, aberrant differentiation and the autoimmune amplification of keratinocytes that ultimately drive psoriatic plaque formation. However, innate immune responses in keratinocytes or plasmacytoid dendritic cells (pDCs) are believed to play an essential role in initiating early inflammatory events that drive the subsequent adaptive immune response [46,47,48,49].

Psoriasis can often be triggered by skin injury, a process known as the “Koebner phenomenon” [50], and psoriasis shares many immunological similarities with the persistent wounding responses [51], indicating that these two pathological events are tightly linked. Similar to wound-activated keratinocytes, suprabasal keratinocytes in psoriatic skin lesions also express high levels of KRT6, 16 and 17, which is considered to be a hallmark of psoriasis [52,53,54]. Recent studies have demonstrated that an elevated expression of these keratins in psoriatic keratinocytes also play a direct role in regulating keratinocyte proliferation, migration and inflammatory responses during the pathogenesis of psoriasis. Therefore, KRT6, K16 and K17 are generally considered the biomarkers and the potential therapeutic targets of psoriasis. Genome-wide association studies connect a range of mutations of keratins to the pathogenesis of psoriasis. Recent studies have identified mutations in K14, K10, K16 and K17 in psoriasis patients [55]. These deleterious mutations are mainly clustered in the rod domain of the keratins, which severely affects the stability of these proteins and causes the changes in keratin expression. Although the mutations of keratins in psoriasis are not fully investigated, those findings offer a clue to better understanding the underlying pathogenesis of psoriasis.

### 2.4. Regulation of Keratin 6/16/17 Expression in Skin Wounds and in Psoriasis

Keratinocytes, positioned directly at the interface with the external environment, have evolved to rapidly sense a variety of external stimuli through surface expression of innate immune pattern recognition receptors (PRRs) (such as Toll like receptors(TLRs), RIG1-like receptor (RLRs) and NOD-like receptors (NLRs-inflammasome)), growth factor receptors (such as epidermal growth factor receptor (EGFR)) and cytokine receptors (interleukin 1 receptor (IL1R) and interferon-alpha receptor (IFNAR)) [38,46,48,56,57,58,59]. During skin injury, necrotic keratinocytes release DAMPs, such as double-stranded RNA, DNA, IL1β, proteins, glycans and lipid or lipoproteins [48,59,60,61,62]. These DAMPs activate keratinocytes through their cognate receptors, such as TLR3 and RIG1-MAVS by dsRNA and IL1R by IL1β, to initiate the inflammatory cascade and the repair pathways [48,62].

**Figure 2:**
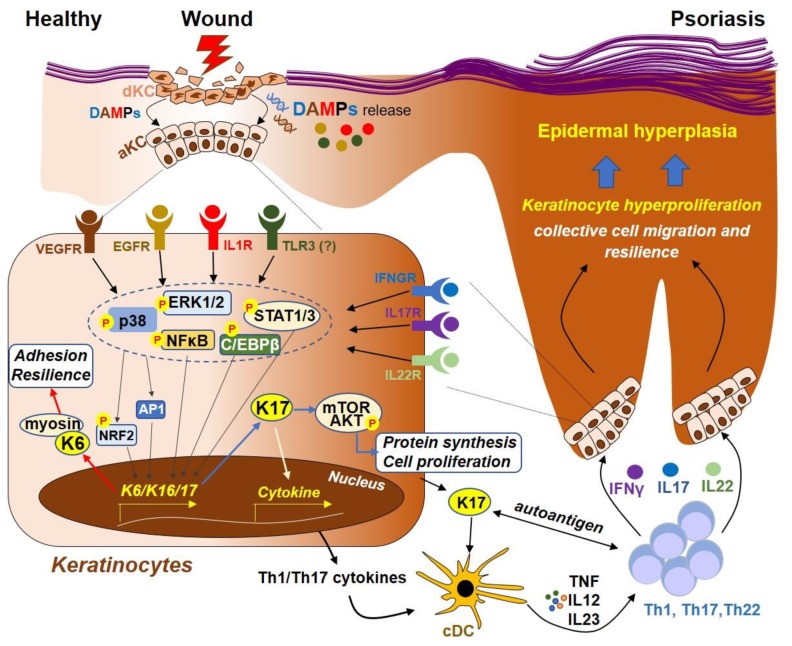
Model for the regulation of Keratin 6, 16 and 17 during skin wounding and in psoriasis. During skin injury, damaged keratinocytes (KCs) release a variety of damage-associated molecular patterns (DAMPs), such as nucleic acids (RNA or DNA) and proteins (such as IL1β). Recognition of these DAMPs by their cognate receptors, such as IL1β by IL1R, EGF by EGFR, dsRNA by TLR3 and/or VEGF by VEGFR, lead to the activation of downstream signaling molecules, including MAPKs (ERK1/2 and p38) and transcription factors CEBPβ, NFκB, NRF2 and AP1, which then translocate to the nucleus and mediate the transcription of K6, 16 and 17. K6 protein can interact with myosin IIA, promoting cell adhesion and resilience that is necessary for KCs to resist mechanical stress during wounding. Furthermore, the K17 protein can promote protein synthesis and cell proliferation by activating the mTOR-AKT pathway. K17 can also translocate to the nucleus, regulating the expression of proinflammatory cytokines through chromatin binding and transcription. In addition, peptides derived from K17 can function as autoantigens to activate conventional DCs (cDC), which then secrete cytokines (TNF, IL12 and IL23) that promote Th1, Th17 and/or Th22 T cell activation. T cell-derived cytokines, including IFNγ, IL17A and IL22, can in turn act on their respective cell receptors that are expressed on keratinocytes, leading to activation of the ERK1/2 and STAT1/3, which further increases the expression of keratin genes, forming an autoimmune positive feedback loop that ultimately drives psoriasis pathogenesis.

Recent studies have identified a complex regulatory mechanism underlying the transcriptional control of KRT6, 16 and 17 during wounding and/or psoriasis (Figure 2). Stimulation of human keratinocytes with active IL1β or EGF, which activates the NFκB and/or MAPK (including ERK1/2 and p38)-AP1 signaling cascades, strongly induces KRT6 or KRT17 expression [63,64,65]. Keratinocytes store an abundant amount of pro-IL1β in an inactive form, and NLR-inflammasome-mediated activation of caspase1 mediates the cleavage of the pro-IL1β to an active form, which is then secreted during UV-irradiation or an inflammatory condition [66,67]. Promoter and gel shift studies have identified AP1, NFκB and C/EBPβ binding sites on the promoter of KRT6, and activation of these transcription factors has been shown to be involved in the transcriptional regulation of KRT6 in response to IL1β and/or EGF [63,64]. These studies suggest that the inflammasome-mediated IL1β secretion may play a role in the induction KRT6 during skin injury. However, whether the wound-related KRT6 expression is also mediated by DAMP-mediated activation of the PRR signaling pathways remains unclear. Furthermore, because AP1 and NFκB are also well-established signaling molecules downstream of TLR3 [68], it is likely that dsRNA released from damaged cells may also contribute to the induction of KRT6 through the TLR3-AP1-NFκB pathway during skin injury.

Studies from Dr. Wang’s group has established an essential role of T cell-derived cytokines, including IFNγ, IL17A and IL22, in maintaining KRT6/16 and KRT17 expression in the chronic lesion of psoriasis [52,69,70,71,72] (Figure 2). Treating human keratinocytes or mouse skin with recombinant IL17A, IFNγ and/or IL22 robustly induces KRT17 expression in epidermal cells, and this is mediated via the STAT1/3 and/or ERK1/2 signaling pathways [69,70]. KRT17 has been identified as a target gene that is suppressed by the TGFBR-SMAD2/3 pathway, and the loss of TGFBR1 expression in psoriatic epidermis contributes to elevated KRT17 expression in psoriasis [73]. Studies have shown that transcription factor Nrf2 also plays a role in controlling KRT6, 16 and 17 expression [72,74]. Nrf2, a transcription factor that is overexpressed in keratinocytes in skin cancer, ichthyosis skin diseases and in psoriasis, has been identified as an important factor promoting keratinocyte hyperproliferation through inducing KRT17 and KRT16 [72,74]. Nrf2 is highly expressed and appears highly activated in the psoriatic epidermis, and Th1/Th17 cytokines promote activation and nuclear translocation of Nrf2, which then binds to the antioxidant responsive element (ARE) on the promoter of keratin genes and mediates the subsequent induction of KRT 6/16/17 in human keratinocyte cell line [72]. Gallic acid (GA), a natural small molecule from *Radix Paeoniae Rubra* with an anti-psoriatic effect, has been shown to suppress IL17A-mediated KRT16 and KRT17 expression by preventing activation of Nrf2 in vitro and in the IMQ-psoriasis mouse model in vivo [74].

Together, the expression of KRT6, 16 and 17 are regulated by a complex network involving several distinct receptor–ligand mediated signaling cascades (as shown in Figure 2). The fact that KRT6, 16, and 17 transcript levels are rapidly induced in wound-proximal keratinocytes within hours after injury in vivo or in the ex vivo model using human skin explants in the absence of T cells [48], suggesting that the initial induction of these keratins during wounding are likely to be triggered by innate immune activation of keratinocytes in response to DAMPs or cytokines, such as dsRNA and IL1β, released locally by skin resident cells. Instead, activated Th1 and Th17 T cells that are recruited later during psoriasis pathogenesis may be necessary to maintain KRT6/16/17 expression during the chronic phase of psoriasis or skin inflammation.

## 3. Keratin 6, 16 and 17—Key Barrier Alarmins During Skin Wounding and/or Psoriasis Pathogenesis

Beyond their well-established role in maintaining the integrity of skin and providing resilience against physical and mechanical stress, keratins have now been recognized as powerful regulators of various cellular functions, such as cell proliferation, migration, differentiation as well as inflammatory and immune responses. The underlying mechanisms that regulate the keratin network and the relevant signaling pathways are highly complex. We next reviewed the roles of KRT6, 16 and 17 in regulating keratinocyte cellular behavior as well as in activating immune responses during skin wounding and psoriasis.

### 3.1. Keratin 6 and 16 Maintain the Cell Adhesion and Optimal Cell Migration During Skin Wounding

Studies from Coulombe’s and other groups using null mouse models has established the role of KRT6 or KRT16 in maintaining cell adhesion and optimal cell migration. Mice with germline deletion of Krt6a and Krt6b, the two isoforms of Krt6, appear normal at birth, but die during the first week after birth due to massive oral epithelial blistering, which results in death due to poor nutrition [75]. Mice overexpressing human KRT16 develop skin lesions concomitant with alterations in keratin filament organization and in cell adhesion at one week after birth [76,77]. In an ex vivo skin explant culture model, Krt6α/β null keratinocytes exhibited an enhanced epithelialization potential due to increased migration [78]. Loss of KRT6α/β was accompanied by a decrease in KRT16 protein expression without a change in K16 mRNA levels, suggesting that K6 may have been necessary for maintaining the stability of its keratin binding partner KRT16 [78]. However, when Krt6α/β null skin was grafted onto immunocompromised mice, Krt6 null KCs exhibited epithelial fragility after incisional wounding, during which swelling and lysis of the null keratinocytes was observed. In addition, this phenotype became exacerbated when the grafted skin was first subjected to chemical irritation followed by mechanical wounding [78]. In a different study, overexpressing human KRT16 in mice led to a delay in wound closure in vivo, and the transgenic K16 skin explants also exhibited a significant reduction in keratinocytes outgrowth [79]. KRT6 interacts directly with Src kinase, dampening its kinase activity and the migratory potential of keratinocytes during wounding [80]. A recent study from Coulombe’s group showed that KRT6 interacts with myosin IIA to limit migration potential of keratinocytes, and KRT6 is also required to maintain the expression of desmoplakin, which mediates the attachment of IFs to desmosomes, to maintain keratinocytes’ cell-cell and cell-matrix adhesion [21]. Together, these results suggest that the KRT6-KRT16 keratin pair may be required for maintaining the resilience necessary to withstand the rigors of a wound site at the cost of a delay in epithelialization, and thus highlights the role of these keratins in collective cell migration.

### 3.2. Keratin 17 Promotes Cell Proliferation

Studies have shown that KRT17, but not KRT6 or KRT16, plays a role in driving keratinocyte hyperproliferation during wounding or psoriasis. Induction of KRT6, KRT16 and/or KRT17 during wound closure is known to occur at the expense of differentiation marker KRT10 and KRT1. Culombe’s group showed that the capacity of KCs to enact this switch was already acquired in mouse embryos at E11.5 [81], a time that was well ahead of the onset of differentiation (~E13.5) and epidermal barrier formation (~E16.5). In addition, only KRT17 null embryos, but not KRT6 null embryos, exhibited significant delay in wound closure compared to WT controls [81]. The lack of a wound closure phenotype in KRT6 null embryos may be due to the concomitant upregulation of the expression of related type II KRT5, which may have functional redundancy to KRT6 [81].

KRT17 null keratinocytes are smaller in size than wild-type cells in vivo and in culture, and protein translation is depressed in KRT17 null cells, correlating with decreased mTOR/AKT signaling, which is essential for cell growth and protein synthesis [82]. It has also been shown that the KRT17 intermediate filament network interacts with STAT3 to facilitate STAT3 phosphorylation and nuclear transportation and induces cyclin D1 expression, leading to keratinocyte hyperproliferation [83]. The absence of KRT17 delays the onset of epidermal hyperplasia, which is preceded by reduced inflammation and a shift of cytokine profiles from a Th1-Th17 dominant profile to a Th2 dominant profile in the mouse model of basal cell carcinoma [84]. This study suggests that KRT17 promotes keratinocyte proliferation by polarizing the immune response towards Th1 and Th17 dominated profile, which is known to boost keratinocyte hyperproliferation.

### 3.3. Keratin 17 Promotes Th1/Th17 Cytokine Production from Keratinocytes

KRT17 is ectopically expressed in numerous pathological skin conditions associated with robust inflammation, including wounds, various skin tumors, virus-induced warts and psoriasis [52,84], suggesting that KRT17 may also play an important role in immune regulation. Studies have demonstrated that KRT17 modulates the expression of an array of inflammatory cytokines that shapes the development of the adaptive T cell responses.

It has been reported that KRT17 interacts with the RNA-binding protein hnRNP K, a member of the heterogeneous nuclear ribo-nucleoprotein family of DNA/RNA-binding protein involved in gene expression [85]. This interaction is required for cytoplasmic localization of hnRNP K and its binding to an array of mRNAs encoding for CXCR3 ligands, such as CXCL9, CXCL10 and CXCL11, and the subsequent expression of these proinflammation mRNAs [85], suggesting a role for KRT17 in RNA export and/or processing. Using a high resolution microscopic technique, recent studies have also identified KRT17 inside the nucleus of tumor epithelial cells [86,87]. Nuclear KRT17 is found to be associated with promoter regions of proinflammatory cytokines, such as CXCL5/10/11, CCL2/19 and IFNG, and KRT17 is also associated with the transcription regulator AIRE and NFκB, indicating the role of KRT17 in chromatin binding and transcription.

### 3.4. KRT17 Peptides Are Auto-Antigens for Psoriatic T Cells

Several peptides derived from the KRT17 protein have been identified to serve as an autoantigen to stimulate T cells in psoriasis, and these KRT peptides are now recognized as the major target antigens recognized by autoreactive T cells isolated from psoriasis patients [52,88,89]. KRT17 peptides share similar epitopes with virulent factors derived from group A Streptococci, a bacterium that often triggers or exacerbates psoriasis [52,88,89]. These antigenic peptides can be first captured by immature dendritic cells (DCs) and subsequently trigger DC maturation and migration to lymphoid organs, where DCs present antigens to and activate both CD4+ and CD8+ T cells [52,88]. Upon contact with these autoreactive T cells in the skin, KRT17 peptides stimulate T cell proliferation and IFNγ production in psoriasis. However, it is still not clear how KRT17 peptides are processed and released from keratinocytes. Future studies will be needed to identify the protease and related secretory mechanisms that are responsible for the release of these autoantigenic KRT17 peptides from psoriatic keratinocytes.

## 4. Therapeutic Potential of Keratins in Skin Diseases

### 4.1. Therapeutic Effect of Keratins in Promoting Wound Repair

As mentioned earlier, keratins are among the first wave of alarmins produced by keratinocytes during wound healing, and these keratins play a vital role in wound repair by providing a framework for cell anchoring, resilience against mechanical trauma, regulating keratinocyte proliferation/differentiation and inflammatory immune responses. Microarray analysis of nonhealing chronic wounds identified KRT16, KRT17, KRT14 and KRT6B among the top 15 downregulated genes in chronic wounds compared to normal skin [90], suggesting that dysregulation of these keratins contributes to defective healing. Therefore, keratin supplementation holds great promise in promoting wound healing, and keratin scaffolds have now received extensive investigations as a new biomaterial for skin regeneration. Keratin dressings have been shown to accelerate wound closure and epithelialization possibly by enhancing the activation of keratinocytes in mice [91,92]. More recently, scientists found that the addition of human adipose stem cells to keratin scaffolds could further accelerate skin wound healing, re-epithelialization and tissue remodeling [93]. Together, the keratin scaffold represents a new natural biomaterial with great therapeutic potential for wound healing. Future studies are needed to determine the exact molecular mechanisms involved in the wound healing process.

### 4.2. KRT17 as A Potential Therapeutic Target for Psoriasis

Conventional treatment options for psoriasis, such as topical corticosteroids, topical vitamin A or D analogues, systemic methotrexate (antimetabolite drug) or cyclosporine therapies and UVB phototherapy, are associated with a broadband immunosuppressive effect and/or organ toxicities, which can cause severe health problems with long term use [94]. New biological therapies have gained popularity due to increased safety and effectiveness compared to conventional therapies. These FDA-approved biological drugs include monoclonal antibodies or inhibitors against TNFα (etanercept, adalimumab and infliximab), IL12 and IL23 (Ustekinumab), IL17A (Secukinumab and Ixekizumab), IL17 receptor A (Brodalumab) and IL23 (Guselkumab, Tildrakizumab, and Risankizumab) [47]. In general, the majority of these psoriasis therapies have been reported to reduce KRT17 expression in keratinocytes [52], suggesting that KRT17 may be critical to psoriasis pathogenesis, and therefore a potential target for therapy. Indeed, knock downing of KRT17 by KRT17-specific antisense oligonucleotides or siRNA inhibits proliferation in keratinocytes in vitro and it is effective in reducing epidermal hyperplasia and inflammatory cell infiltration on transplanted psoriatic tissue in a mouse model [71]. Together, KRT17 holds great potential as a new target for the treatment of psoriasis in the future.

### 4.3. Targeting KRT6 for Pachyonychia Congenita

Pachyonychia congenita (thick nails, plantar keratoderma), a rare keratinizing, autosomal dominant inherited skin disorder, is caused by mutations in KRT6, 16 and 17 genes [7]. In clinical trials, KRT6a N171K mutant siRNA, which specifically and potently targets the mutant mRNA without affecting wildtype KRT6a mRNA, has been shown to have clinical efficacy in treating pachyonychia congenita [95]. The development of keratin mutant allele-specific interventions holds great potential not only for treating pachyonychia congenita, but also for treating other inflammatory skin disorders caused by autosomal mutations in keratin genes.

## 5. Conclusions

As a critical barrier, the skin has the ability to rapidly repair wounds after physical trauma, and recent studies have demonstrated that keratins, the major structural intermediate filaments in keratinocytes, play vital roles during the healing process. KRT6, 16 and 17 have been recognized as critical barrier alarmins that are rapidly induced during the first wave of wound response in keratinocytes. The induction of these keratins is regulated by a highly complex signaling network involving activation of several receptors by DAMPs, growth factors, and/or cytokines from activated KCs or T cells. These keratins contribute to wound healing by providing keratinocytes with optimal cell adhesiveness, directionality, mechanical integrity/resilience against physical stress and proliferative potential that ultimately leads to collective cell migration and regeneration during wound repair. Among these keratins, KRT17 can directly stimulate cell proliferation, and inflammatory cytokine production from keratinocytes. In addition, KRT17 peptides can also serve as autoantigens to activate APC-T cells, leading to the generation of the pathological auto-reactive T cells that drive psoriasis. Together, these cytoskeletal proteins play a fundamental role in facilitating dynamic and tightly regulated signaling cascades during wound repair and in psoriasis pathogenesis, and are great potential therapeutic targets for the treatment of defective wound healing and inflammatory skin disorders such as psoriasis.
